# Evidence of competition between electrogens shaping electroactive microbial communities in microbial electrolysis cells

**DOI:** 10.3389/fmicb.2022.959211

**Published:** 2022-12-16

**Authors:** Marie Abadikhah, Miguel de Celis Rodriguez, Frank Persson, Britt-Marie Wilén, Anne Farewell, Oskar Modin

**Affiliations:** ^1^Division of Water Environment Technology, Department of Architecture and Civil Engineering, Chalmers University of Technology, Gothenburg, Sweden; ^2^Department of Genetics, Physiology and Microbiology, Complutense University of Madrid, Madrid, Spain; ^3^Institute of Chemistry and Molecular Biology and the Center for Antibiotic Resistance Research, University of Gothenburg, Gothenburg, Sweden

**Keywords:** bioelectrochemical system (BES), bioanode, biocathode, microbial electrolysis cells (MECs), microbial community assembly

## Abstract

In single-chamber microbial electrolysis cells (MECs), organic compounds are oxidized at the anode, liberating electrons that are used for hydrogen evolution at the cathode. Microbial communities on the anode and cathode surfaces and in the bulk liquid determine the function of the MEC. The communities are complex, and their assembly processes are poorly understood. We investigated MEC performance and community composition in nine MECs with a carbon cloth anode and a cathode of carbon nanoparticles, titanium, or stainless steel. Differences in lag time during the startup of replicate MECs suggested that the initial colonization by electrogenic bacteria was stochastic. A network analysis revealed negative correlations between different putatively electrogenic *Deltaproteobacteria* on the anode. Proximity to the conductive anode surface is important for electrogens, so the competition for space could explain the observed negative correlations. The cathode communities were dominated by hydrogen-utilizing taxa such as *Methanobacterium* and had a much lower proportion of negative correlations than the anodes. This could be explained by the diffusion of hydrogen throughout the cathode biofilms, reducing the need to compete for space.

## 1. Introduction

Microbial electrochemical technologies (METs), such as microbial fuel cells and microbial electrolysis cells (MECs), have many potential applications within environmental engineering, including the production of renewable energy, the treatment of wastewater, and recovery of resources such as metals (Modin and Aulenta, 2017). Some of the technologies have been commercialized (Aftab et al., 2020). METs use electroactive microorganisms as catalysts in the breakdown of organic material, resulting in the generation of current (De Vrieze et al., 2018; Mateo et al., 2018). The biofilm on the anode surface oxidizes organic material, resulting in the release of electrons, which are then transported to the cathode where a reduction reaction takes place (Shin et al., 2017; De Vrieze et al., 2018; Logan et al., 2019). The bacteria responsible for the current generation at the anode are usually referred to as electrogenic bacteria, and some of the most well-known genera are *Geobacter* and *Shewanella* (Bond and Lovley, 2003; Shin et al., 2017; Logan et al., 2019). However, anode biofilms are diverse communities with several functional groups of microorganisms in syntrophic interactions (Kokko et al., 2018). On the cathode, *Methanobacterium* spp. are often found to catalyze the reduction in CO_2_ to CH_4_ (Siegert et al., 2015), and several acetogens are known to reduce CO_2_ to acetate with a cathode as the electron donor (Nevin et al., 2011). Cathode communities are less diverse than anode communities (Logan et al., 2019).

The microbial communities on the electrode surfaces are crucial for the function of METs. The performance of electrogenic biofilms is often reported to be dependent on the relative abundance of *Deltaproteobacteria*, particularly *Geobacter* spp. (Yates et al., 2012; Koch et al., 2019). There are, however, variations between replicate reactors operated under identical conditions, which give rise to differences in performance (Zhou et al., 2013). At present, we lack knowledge about why such differences occur. Many factors affect the community composition and function in METs, and changes occur over time (Zhang et al., 2014). Ecological factors shaping microbial communities have been categorized as selection, drift, diversification, and dispersal. Selection refers to differences in fitness between species, which give some species advantages under certain environmental conditions. Drift refers to the random death and replication of species. Diversification refers to processes such as mutations and horizontal gene transfer that led to the evolution of species. Dispersal is the attachment, detachment, and movement of microorganisms (Vellend, 2010; Nemergut et al., 2013). In the case of METs, environmental factors such as the design and material of the system, electrode potentials, and the composition of the substrate will result in the selection of certain species. For example, different electrode materials in microbial fuel cells coupled with constructed wetlands were shown to result in microbial communities with different compositions (Wang et al., 2016), and the anode potential affected the microbial community in propionate-fed MECs (Hari et al., 2016). The type of organic substrate was also shown to have a large effect on the structure of electrogenic communities on anodes in several studies, with more complex substrates leading to more diverse communities (Koch et al., 2019; Saheb-Alam et al., 2019a). Less is known about the roles of drift, diversification, and dispersal for microbial community assembly in METs. These factors contribute to stochastic changes in the microbial community structure. For example, stochastic initial colonization was shown to lead to differing communities and functions of MECs operated under identical conditions (Zhou et al., 2013). The importance of initial colonization for biofilm community development was also shown in a study with two strains of *Shewanella oneidensis* (Kees et al., 2021). Other studies suggested that deterministic factors (selection) lead to converging microbial communities over time (Yates et al., 2012).

In single-chamber systems, the anode and cathode are placed in the same compartment. Such systems are often used for the production of hydrogen or methane (Li et al., 2019). Different biofilms form on the anode and the cathode, and there can also be microorganisms suspended in the liquid. Each location within the system (anode, cathode, and liquid) has specific environmental conditions that select certain species. However, a continuous dispersal of microorganisms could affect the community composition at each location. There is also a functional connection between the different locations. For example, a well-functioning cathode will enable higher current generation in the system, which affects the selection pressure at the anode. The overall rate of reactions in the system will also determine the concentrations of metabolites, which will affect selection pressures in all locations.

The aim of this study was to investigate the effect of different cathode materials on the microbial communities within single-chamber MECs. Carbon nanoparticles were chosen for their large surface area, titanium has previously been used as a cathode for metal recovery in MECs (Modin et al., 2012), and steel was chosen due to its use as cathode material for hydrogen production in MECs (Wang et al., 2018). We hypothesized that the cathode material would not only affect the community at the cathode but also the community at the anode and in the liquid; we wanted to compare the deterministic effect of the cathode material with the stochastic effects of dispersal and drift. This was done by operating replicate MECs where only the cathode material was different and by observing system performance and microbial community composition. The three cathode materials tested – carbon nanoparticles, titanium, and steel – did not drive differences in microbial community composition between the systems. Instead, we found that intra-genus competitions both within electrogenic *Deltaproteobacteria* at the anode and methanogenic archaea at the cathode occurred and explained part of the observed differences in microbial communities between the nine MECs.

## 2. Materials and methods

### 2.1. MECs

**Figure 1:**
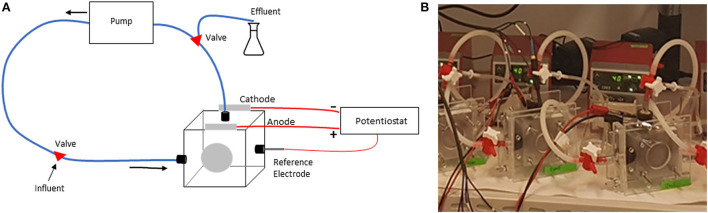
**(A)** A schematic illustration of the reactor setup, **(B)** a photo of the reactor setups in the lab.

Nine single-chamber MECs were constructed from Plexiglas (Figure 1). The MEC was connected to a peristaltic pump using PVC tubing (4-mm diameter). The anode in all MECs was made by pressing a carbon cloth (AvCarb 1071 HCB, Fuelcellearth.com) against a carbon foil support layer (Alfa Aesar). Three cathode materials were used in replicates of three: Carbon paper coated with carbon nanoparticles, titanium foil (Sigma Aldrich), and stainless steel (EN 1.4301). The carbon paper (AvCarb P75T, Fuelcellearth.com) with carbon nanoparticles was prepared by vortexing a mixture of Cabot Black Pearls 2000 (0.15 mg/cm^2^), PTFE (0.05 mg/cm^2^), and propanol (30 μl/cm^2^). The mixture was then evenly painted onto the carbon paper surface, air dried, and finally heated in the oven for 20 min at 350°C for the PTFE to melt and bind the nanoparticles to the carbon paper. The system was connected to an effluent tube containing a nutrient medium, which minimized oxygen back-diffusion into the MEC. The three replicate MECs with carbon nanoparticle cathodes are labeled as C1, C2, and C3; and three with titanium cathodes as T1, T2, and T3; and the three with steel cathodes as S1, S2, and S3.

### 2.2. MEC operation

The nutrient medium (NM), consisting of 0.1 g/L KCl, 0.6 g/L KH_2_PO_4_, 0.25 g/L NH_4_Cl, 3 g/L NaHCO_3_, 0.1 g MgCl_2_, and 0.03 g/L CaCl_2_, was prepared according to the instructions by Saheb-Alam et al. (2018), and the trace mineral solution was prepared according to the instructions by Marshall et al. (2012). The NM was supplemented with organic carbon in the form of sodium acetate (0.60 g/L), sodium propionate (0.40 g/L), and sodium butyrate (0.32 g/L) before being added to the MECs. The total volume of each MEC including the tubing was 70 ml. The fluid was circulated at a flow rate of 40 ml/min. Before inoculation, the MECs were rinsed with deionized water followed by the NM. The MECs were inoculated with 5 ml of mesophilic anerobic digester sludge and operated for a total of 104 days at room temperature (~20°C). The anerobic digester sludge was prepared for inoculation by mixing to homogenize the sludge before sampling. During regular operation, 50 ml of the NM media within the reactors was replaced at regular intervals of 2–3 days, and samples were taken from the effluent. A cell potential of 1 V was kept between the anode and cathode using a potentiostat (MultiEmStat3+, PalmSens). At this cell potential, no current generation was observed before an electrogenic microbial community had been enriched.

In specific tests, 5 ml samples were taken at 0, 24, and 72 h after feeding and replaced with 5 ml NM media with only a single carbon source of acetate, propionate, or butyrate to investigate the transformation of that particular substrate. These tests were performed between day 72 and day 88.

### 2.3. Analytical methods

The concentrations of acetate, propionate, and butyrate in the samples were determined using a high-performance liquid chromatograph (HPLC) with a UV detector (Shimadzu) and an Aminex HPX-87H Ion Exclusion column (BioRad). The bioelectrochemical activity of the electrodes was examined using cyclic voltammetry (CV). A scan rate of 5 mV/s was used, and three repeated scans were performed each time. Ag/AgCl reference electrodes (BAS Inc.) with an offset against the standard hydrogen electrode (SHE) of +197 mV were used. All electrode potentials in the study are reported against the SHE. Polarization curves with the cell potential changed at a scan rate of 5 mV/s were also determined.

### 2.4. Calculations

The current generated in the MECs was measured every 30 s using the potentiostat. Before further analysis, groups of ten current measurements were averaged into values representing 5-min intervals. The peak current for a time period (e.g., a batch cycle) was the highest 5-min average current observed during the period. The total charge for a given time period was calculated by integrating the current generation over time (Equation 1). The coulombic efficiency is the fraction of the removed organic compounds that result in current production (Equation 2):


(1) $$\begin{array}{l} Total\;charge=\int_{t_{1}}^{t_{2}}I\left(t\right)\cdot dt \end{array}$$

(2) $$\begin{array}{l} \;Coulombic\;efficiency\\ =\frac{\int_{t_{1}}^{t_{2}}I\left(t\right)\cdot dt}{F\cdot V\cdot \left({b_{ac}\cdot \Delta C}_{ac}+{b_{prop}\cdot \Delta C}_{prop}+{b_{but}\cdot \Delta C}_{but}\right)}, \end{array}$$

where *I* is the current (A), *t* is time (s), *F* is Faraday's constant (96,485.3 C/mol e^−^), *V* is the liquid volume in the MEC (L), Δ*C* is the change in concentration of the substrate acetate, propionate, and butyrate (mol/L), and *b* is the number of electrons liberated when the substrates are oxidized to CO_2_ (mol e^−^/mol substrate).

### 2.5. Microbial community analysis

The inoculum, biofilms on the anode and cathode, and the biomass suspended in the liquid in each MEC were sampled at the end of the experiment. Biofilm growing in the effluent tube in three of the MECs was also sampled. In addition, the foam produced in two of the MECs (C2 and S3) during CV tests with malfunctioning reference electrodes was sampled at the time of the CV measurements. The samples were stored at −20°C until DNA extraction. The titanium and steel cathode biofilms were extracted using a sterile spoon to scrape the biofilm from the surface of the material. For the CNP cathodes and all anode biofilms, a sterile scissor was used to cut the material into smaller fragments. DNA was extracted using the FastDNA Spin kit for Soil (MP Biomedicals). The protocol for DNA extraction was followed apart from the homogenization step, which was repeated one time. The V4 region of the 16S rRNA gene was amplified using the primer pair 515'F (GTGBCAGCMGCCGCGGTAA) and 806R (GGACTACHVGGGTWTCTAAT) (Caporaso et al., 2011; Hugerth et al., 2014). The Phusion master mix (40 μl) was combined with 2 μl of the sample, reverse and forward primers, respectively. The Phusion master mix consists of 8 μl 5 × Phusion Buffer, 0.8 μl 10 mM dNTPs, 1.2 μl DMSO, 0.4 μl Phusion hot start II polymerase, and 23.6 μl ultrapure water (ThermoFisher Scientific). The PCR was done with a Bio-Rad T100 Thermal Cycler with the settings: activation at 98°C for 30 s, 34 cycles of denaturation for 10 s at 98°C, annealing for 30 s at 55.8°C, and extension at 72°C for 30 s. This was then followed by the final elongation for 10 min at 72°C. The PCR product was purified (MagJET NGS Cleanup and Size Selection Kit, ThermoFischer Scientific), and the DNA concentration was measured using a Qubit Fluorometer (Thermo Fisher Scientific). The samples were then pooled before sequencing using an Illumina Miseq system with the Miseq reagent kit v3 and 2 × 300 bp read length. The sequence data were processed using VSEARCH (Rognes et al., 2016) and DADA2 (Callahan et al., 2016). A consensus count table consisting of the amplicon sequence variants (ASVs) detected with both methods was constructed using qdiv (Modin et al., 2020). Taxonomic classification of the ASVs was done using the Midas database (Nierychlo et al., 2020). Due to a low read count, cathode sample T2 was excluded from the microbial community analysis and the network analysis.

Microbial diversity was calculated in qdiv using the Hill-based framework for alpha and beta diversity (Chao et al., 2014; Modin et al., 2020). Beta diversity was visualized using a principal coordinate analysis (PCoA). A one-way single ANOVA and a *post-hoc* test (Tukey's HSD) were conducted using Pinguoin (Vallat, 2018). To determine whether differences in community composition could be explained by random chance, the Raup–Crick null model was used with 999 iterations (Raup and Crick, 1979; Modin et al., 2020). To examine correlations between differences in community composition and differences in system performance, the Mantel test was used (Mantel, 1967). The raw sequence reads have been deposited in NCBI's Sequence Read Archive (SRA) and are accessible through the BioProject accession number PRJNA839919.

### 2.6. Network analysis

For the network analysis, anodes and cathode samples were analyzed separately. Samples were filtered so that only ASVs present in at least two samples within the group and with a relative abundance higher than 0.1% in at least one sample were retained. The phylogenetic distances of ASVs were calculated to combine pairs of ASVs that were phylogenetically close. For this, a maximum likelihood tree was constructed using the GTR+G+I model with the phangorn R package (Schliep, 2010) using sequences aligned with the msa R package (Bodenhofer et al., 2015). Then, the distance between each pair of tips from the phylogenetic tree using its branch lengths was calculated, with the cophenetic function of the ape R package (Paradis and Schliep, 2018). This resulted in a total of 146, 113, and 299 ASVs for the anode, cathode, and suspension microbial communities, respectively. Every potential co-occurrence and co-exclusion between nodes was calculated by applying two correlation models, Spearman's rank correlation and Sparse Correlations for Compositional data (SparCC) algorithm implemented in the SpiecEasi R package (Kurtz et al., 2015). The co-occurrences/co-exclusions were considered valid if Spearman's correlation coefficient (ρ) and SparCC R-corr absolute values were higher than 0.6 and if they were statistically significant with *p*-values lower than 0.05. The networks were visualized using the igraph R package (Csardi and Nepusz, 2006).

## 3. Results

### 3.1. Replicate MECs with varying start-up times

**Figure 2:**
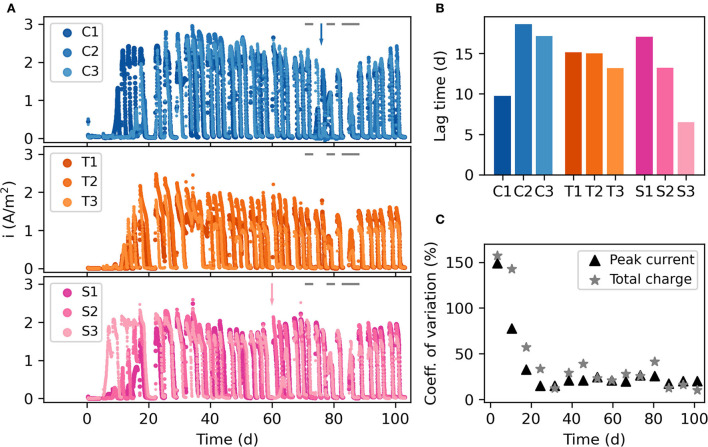
**(A)** Current density throughout the experimental run. The gap around day 85 is when the three-day experiment with no current was performed. C1–C3 are the carbon nanoparticle reactors, T1–T3 are the titanium reactors, and S1–S3 are the steel reactors. The arrows show when foaming incidents occurred in C2 and S3. The gray bars at the top of the panels show when specific tests with individual carbon sources were carried out. **(B)** Lag time before each MEC started producing current. **(C)** Variation in peak current and total charge per week for all 9 reactors.

Triplicate reactors were set up with three different cathode materials, carbon nanoparticles (C1-3), titanium (T1-3), and steel (S1-3). Figure 2A illustrates the current generated during the experiment. As the MECs were fed batchwise every 2–3 days, there was an increase in current upon feeding and a decrease when electron donors were depleted in each batch cycle. The lag time before substantial current production (>1 A/m^2^) began at the start of the experiment varied between 5 and 17 days (Figure 2B). There was no clear difference in lag time between MECs having different cathode materials, although the titanium MECs had the smallest difference among the replicates. Because of the difference in start-up times, there was a large variation in peak current and total charge generated by the nine MECs during the first 3 weeks of the experiment (Figure 2C).

### 3.2. No significant difference in current production and coulombic efficiency between MECs with different cathode materials

Once the MECs started producing current, a gradual increase was observed until they all reached their peak current generation near day 30. After this point, the current generation decreased slightly until a stable level was obtained (Figure 2A; Supplementary Figure S1). The coulombic efficiency had a clear peak near day 30, reaching between 33% (T3) and 56% (C2). It then decreased to around 20% in all MECs (Supplementary Figure S1). There was no statistically significant difference in peak current and coulombic efficiency between the MECs with different cathodes (*p* > 0.05, ANOVA), except the week around day 52 when the carbon nanoparticle MECs generated higher currents and more charge. At day 60, a malfunctioning CV test in S3 resulted in no current generation for 5 days in that MEC (Figure 2A).

### 3.3. Bioelectrochemical catalysis of anode and cathode reactions improved with biofilm development, and there were differences between cathode materials

**Figure 3:**
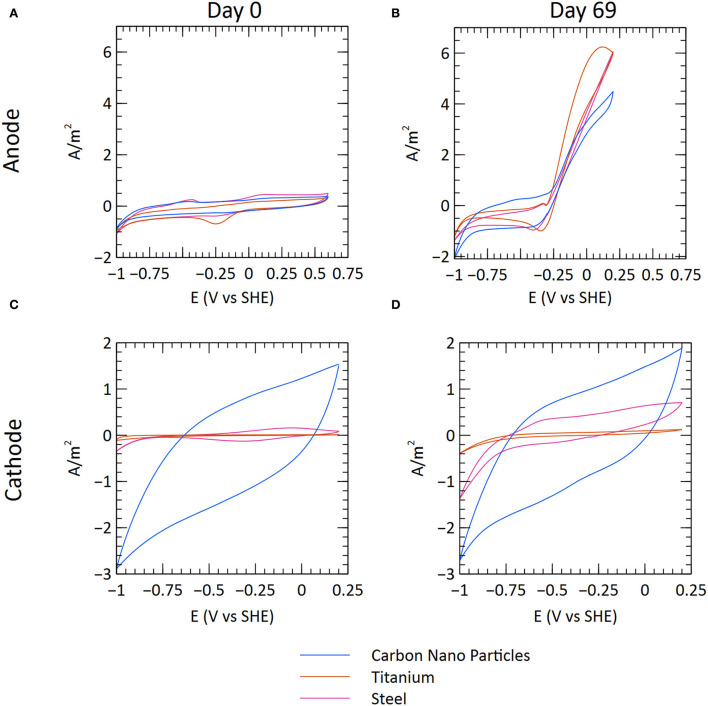
Cyclic voltammetry measurements from the start (after inoculation) and end of the experiment. The graphs illustrate a representative MEC for each material (C1, T3, and S2). **(A)** Anode at the start of the experimental run. **(B)** Anode at the end of the experimental run. **(C)** Cathode at the start of the experimental run. **(D)** Cathode at the end of the experimental run.

CV was performed on several occasions to characterize the electrochemical properties of the systems. A clear difference can be seen when comparing the start and end of the experiment, indicating that changes occurred due to the growth of microbial communities on the electrode surfaces (Figure 3; Supplementary Figures S3, S4). Both the anodes and the cathodes showed evidence of bioelectrochemical catalysis. The anodes showed a rapid rise in the current generation at potentials exceeding −0.25 V vs. SHE. The rise in anodic current for the carbon nanoparticle anodes declined in the CVs carried out on day 76 (Supplementary Figure S3). There was also a lower current generation during this time period of MEC operation (Figure 3), possibly because of specific tests being carried out.

For the cathodes, there were differences between the materials. The increase in cathodic current (negative current) at potentials lower than approximately −0.8 V vs. SHE corresponds to the hydrogen evolution reaction (2H^+^ + 2e^−^ → H_2_). The ability of the steel and titanium electrodes to catalyze hydrogen evolution appears to have improved during the experiment because there is a sharper increase in cathodic current. No clear difference can be seen for the carbon nanoparticle cathodes, which show high hysteresis both at the start and the end of the experiment. Polarization curves showed a linear relationship between current and cell potential, suggesting that ohmic losses limited current generation in the MECs (Supplementary Figure S5).

### 3.4. Current generation was affected by carbon source

Once the current generation was stable within the systems, the effect of the carbon substrate composition was investigated in 72 h tests with only one carbon source in the NM. The highest peak currents were observed with acetate as the only carbon source. With propionate and butyrate as single carbon sources, the peak currents were only about half of those with acetate, and the current generation profiles were more drawn out over time (Supplementary Figure S2). Most of the substrate was consumed within 24 h in all cases. Tests with only acetate or propionate showed a slight increase in butyrate concentrations, while tests with only propionate and butyrate showed a slight increase in acetate concentration (Supplementary Figure S6). There was no clear difference in coulombic efficiency for different carbon sources (Supplementary Figure S7).

### 3.5. Organic carbon was consumed both in the presence and absence of electrical current

On day 83, potentiostatic control of the MECs was stopped for 48 h. During this time, a feeding cycle was carried out to observe the consumption patterns of the three substrates in the absence of an electrical current. A slightly higher acetate concentration was observed after 24 h without current, but there were no major differences in consumption profiles for propionate and butyrate (Supplementary Figure S8).

### 3.6. Malfunctioning reference electrodes disrupted bioelectrochemical activity on two occasions

During two of the CV tests, malfunctioning reference electrodes led to excessive current generation and foam formation in the MECs. This happened in C2 on day 76 and S3 on day 60. After these events, it took 8 days in C2 and 7 days in S3 before the current generation recovered to the same levels as before the malfunctioning event.

### 3.7. Alpha diversity was highest in the suspension and there was a large variation between different MECs

**Figure 4:**
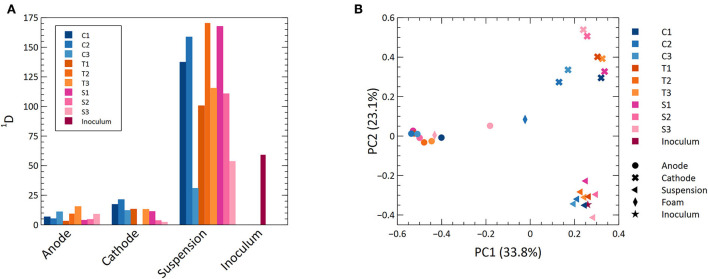
**(A)** A bar graph with the alpha diversity values for all samples at a diversity order of 1. **(B)** Principal coordinate analysis of dissimilarities at a diversity order of 1.

Measurements of microbial diversity were done at the end of the experiment. Alpha diversity measured as the Hill number of diversity order 1 is shown in Figure 4A. This index gives weight to the relative abundance of ASVs (Jost, 2006) and can be interpreted as the number of “common” ASVs in a sample (Chao et al., 2010). The suspension had a higher alpha diversity than the anodes and cathodes (*p* < 0.05, ANOVA and Tukey's HSD). The anode and cathode had more similar values, although the anode had the lowest (*p* > 0.05, ANOVA). There was a large variation in diversity between samples from the same location (i.e., anode, cathode, or suspension) in different MECs (Figure 4A; Supplementary Figure S9a). There was no difference in diversity between MECs having different cathode materials from the same location (*p* > 0.05, ANOVA).

### 3.8. Location within the MEC drives microbial community composition, while the cathode material does not

A PCoA including all samples is shown in Figure 4B. There was a distinct separation based on the location within the MECs showing that different locations represent different habitats for the microorganisms (Figure 4B; Supplementary Figure S9). The inoculum clustered with the suspended biomass. One foam sample clustered with the anodes and the other appeared between anodes and cathodes.

### 3.9. The effect of cathode material on microbial community composition was low in comparison to other factors

A null model analysis was carried out to determine whether the dissimilarity between microbial communities from the same location (e.g., anodes) in different MECs was higher or lower than random chance. The null model fixes the number of ASVs in each community and evaluates compositional dissimilarity. Thus, if the null model identifies significant similarity or dissimilarity between pairs of communities, this is caused by the composition of ASVs, not by similarity or dissimilarity of the number of detected ASVs in the communities. No significant difference in composition between communities from MECs with different cathode materials could be observed for a diversity order of 1, which gives weight to the relative abundance of ASVs. Only the anode in MEC S3 and the cathode in MEC C2 had significantly different composition to some of the anodes and cathodes in the other MECs (Supplementary Table S1). These were the two MECs that experienced foaming incidents. For diversity order 0, there were many significant similarities (^q^RC < 0.05), both in the comparison among anodes and cathodes.

The Mantel test was done to determine whether there was a correlation between the difference in total electrical charge generated in different MECs during the experiment or peak current at the end of the experiment with dissimilarity in microbial community composition between MECs. No significant correlations could be observed (*p* > 0.05).

### 3.10. *Deltaproteobacteria* dominated on the anodes, and methanogens and acetogens dominated on the cathodes

**Figure 5:**
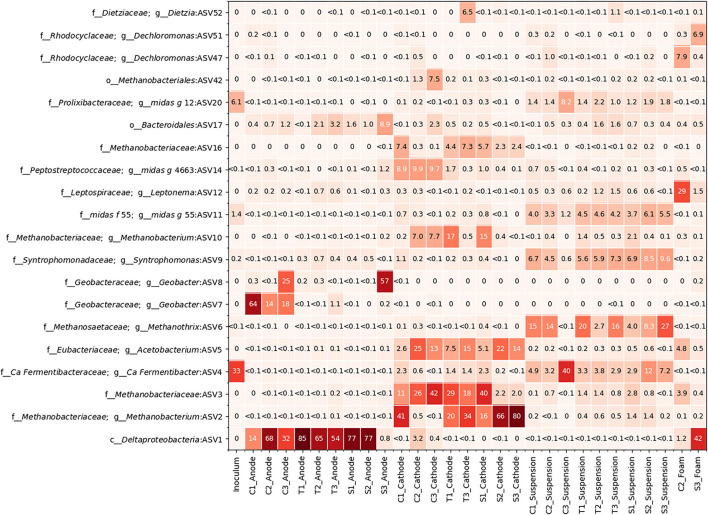
A heatmap depicting the relative abundance of the top 20 most abundant taxa present in the 9 reactors, the inoculum, and the foam samples from C2 and S3.

Figure 5 shows the relative abundance of the most abundant ASVs in the MECs. A *Deltaproteobacteria* sp., unclassified at the genus level, (ASV1) was present in high abundance across all anodes except S3. It was, however, in high abundance in the foam sample taken from S3. A *Geobacter* sp. (ASV7) was present in high abundance on the anodes from C1 to C3. Another *Geobacter* sp. (ASV8) had a high abundance in the anodes from C3 and S3 (Figure 5; Supplementary Figure S10).

All the cathode communities had a high abundance of methanogens (ASV2, ASV3, and ASV10) from the *Methanobacteriaceae* family. S2 and S3 had a very high abundance of ASV2 on their cathodes and a very low abundance of ASV3, while the opposite was true for C2 and C3 (Figure 5; Supplementary Figure S8). *Acetobacterium sp*. (ASV5) was also present on some of the cathodes.

Several fermentative bacteria were found to be present in all locations. *Ca. Fermentibacter* sp. (ASV4) was found in high abundance in both the inoculum and C3 suspension, as well as in varying levels of abundance for the suspensions in most of the other MECs. There was also a high abundance of *Synhtrophomonas* sp. (ASV9) and *Methanothrix* sp. (ASV6) in the suspension. The foam sample from C2 had some *Acetobacterium* sp. (ASV5) and *Methanobacteriaceae* sp. (ASV3) but mainly consisted of *Leptonema sp*. (ASV12) (Figure 5).

The microbial community sample from the effluent tube showed a high abundance of aerobic organisms such as taxa from the *Rhodococcus, Aquimonas, Flavihumibacter*, and *Pseudoxanthomonas* genera (Supplementary material).

### 3.11. Microbial correlation network analysis highlights the competition between different *Deltaproteobacteria* spp. for dominance on the anode surface

**Figure 6:**
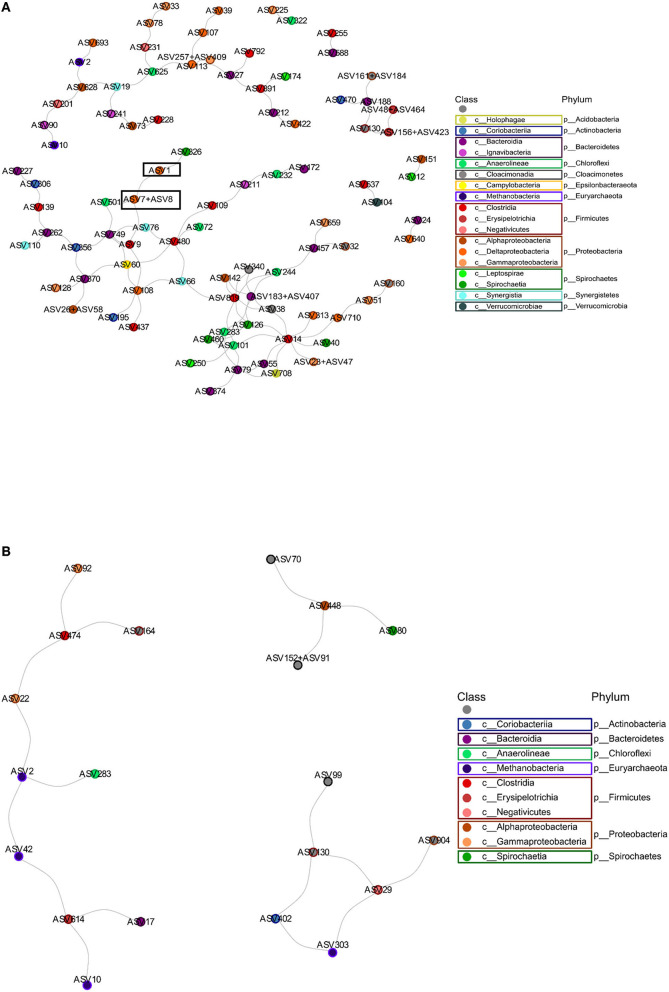
The microbial correlation network depicting the negative interactions. **(A)** The anode microbial community. The interaction between ASV1 and the combination of ASV7 and ASV8 is highlighted. **(B)** The cathode microbial community. Colors depict the taxonomy of each ASV at the class level.

**Figure 7:**
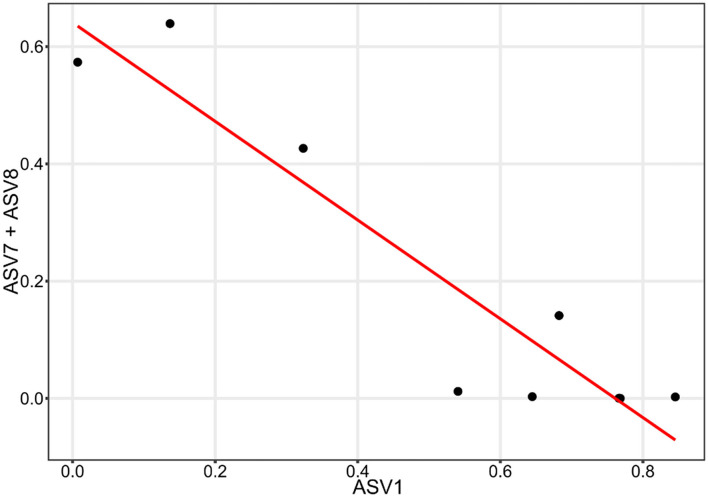
Linear correlation between the abundance of ASV1 and the combined abundance of ASV7 and ASV8.

A microbial correlation network analysis was performed to identify any potential positive or negative interactions between the different taxa in the anode and cathode communities. The interaction network obtained from the anodes was more complex, having a higher number of total edges, as well as a higher percentage of negative edges than the cathode network. The cathode had many fewer negative interactions, consisting of only 7.6% of the 236 interactions determined (Figure 6, Table 1). Some interactions could be seen between ASVs belonging to *Methanobacteria*, e.g., ASV2 and ASV42 (Figures 5, 6B). *Methanobacteria* sp. ASV10 was also present in the same cluster although it did not have a direct negative interaction with either ASV2 or ASV42. Both ASV10 and ASV42 have a negative interaction with ASV614, which is a *Erysipelothrix* sp. (Figure 6B). In comparison, the anode had a total of 337 interactions, out of which 30.0% were negative (Table 1). When ASV7 and ASV8, which only differed by one base pair, were considered as an individual taxon, a negative correlation with ASV1 can be seen (Figures 6A, 7). Negative correlations between ASV113, ASV117, and ASV39 within *Deltaproteobacteria* spp. were also observed on the anode (Figure 6A). Positive interactions could be seen been on the anodes between *Deltaproteobacteria* and some fermentative bacteria. For example, ASV1 had positive correlations with ASV9 and ASV437, which were both in the class *Clostridiales* (Supplementary Figure S12).

**Table 1:** A summary of the network properties for the anode, cathode, and suspension microbial communities.

**Network Property**	**Anode**	**Cathode**	**Suspension**
Number of nodes	133	102	296
Number of edges	337	236	1,224
Positive edges (%)	236 (70.03)	218 (92.37)	1,073 (86.66)
Negative edges (%)	101 (29.97)	18 (7.63)	151 (12.34)
Edge density	0.038	0.046	0.028
Number of modules	19	9	19
Modularity	0.518	0.690	0.612
Clustering	0.357	0.607	0.424

## 4. Discussion

### 4.1. Temporal trends in MEC function

There was no clear effect of cathode materials on the start-up of the MECs. Interestingly, there was a large variation between the replicate reactors in lag time before the current production began. This suggests that initial attachment and colonization of the anode by electrogenic bacteria is a stochastic process. The dominance of stochastic factors during the initial phase of biofilm development was recently shown for anammox biofilms (Niederdorfer et al., 2021) and for community assembly in MECs (Zhou et al., 2013).

Despite differences in the initial start-up, all reactors reached a high current generation and coulombic efficiency between day 25 and day 40, which then declined to a lower but stable level (Figure 2). During the early stages of the experiment, the anode biofilm likely consisted of a thin layer dominated by electrogenic bacteria. The diffusion resistance of organic carbon to the electrogenic bacteria was low, and electrons would be efficiently transferred to the anode surface. As the biofilm grew, non-electrogenic bacteria found niches in the biofilm. This likely limited the electrogenic bacteria's access to organic carbon and nutrients, which had to diffuse through a thicker biofilm. There should also have been a competition for space on the anode surface between electrogenic and non-electrogenic bacteria (Sun et al., 2016). These phenomena may explain the reduction in current density and coulombic efficiency after the peak at day 25–40. This type of transition in biofilm community structure and function has previously been observed on chitin particles in sea water, where the early colonization by primary chitin consumers led to rapid degradation of chitin. Over time, the fraction of secondary consumers degrading, e.g., cell debris and metabolic by-products increased in the biofilm, which led to a lower degradation rate of the primary substrate (Datta et al., 2016; Enke et al., 2018). In our experiment, the decline in current density was less drastic than the decline in coulombic efficiency during the experiment (Supplementary Figure S1). The current density could likely be maintained because of the high concentrations of nutrients, which limited the effect of diffusion resistance. Some bacteria known to be present in these kinds of systems, such as *Geobacter sulfurreducens*, have also been shown to use nanowires and mediators to transfer electrons to the anode (Reguera et al., 2006; Marsili et al., 2008), which means that bacteria located far from the anode surface could still contribute to current generation. It is also possible that interactions between bacteria within the biofilm and the interaction with bacteria located in the area close to the anode surface could play a role in increasing the accessibility to nutrients and electrons for transfer. For example, the association between fermenters and electrogenic bacteria may be needed for current generation from propionate and butyrate. The experiment with each carbon source fed individually to the MEC suggested that the systems responded more slowly to propionate and butyrate than they did to acetate.

The decline in coulombic efficiency after the peak from day 25 to day 40 suggests that alternative carbon utilization pathways developed in the systems over time. Methanogenesis is likely the most important, although aerobic oxidation could contribute because of trace amounts of oxygen leaking into the system. The energy gain for microorganisms carrying out methanogenesis is lower than that for electrogens, which explains why methanogenic consortia using acetate, propionate, and butyrate needed a longer time to establish in the system than electrogens. In a specific test, the applied potential was turned off, which disabled electrogenic activity in the systems. This did not result in major differences in organic carbon consumption rates compared to normal operation, which suggested that non-electrogenic bacteria could rapidly utilize the available resources in the system when electrogenic bacteria were inactive. Acetate was the only substrate with a slightly slower degradation rate without applied potential (Supplementary Figure S8), which could be explained by acetate being the primary substrate for electrogens, while propionate and butyrate were first degraded by fermenters.

Cyclic voltammetry showed that the CNP cathodes had higher catalytic activity for the hydrogen evolution reaction than titanium and steel. However, this did not result in a higher current generation in the MECs with CNP cathodes because the performance of the systems was limited by ohmic losses. Over time, the catalysis of the hydrogen evolution reaction improved on the steel and titanium cathodes, likely a result of biofilm formation. A variety of hydrogenotrophic microorganisms have previously been shown to catalyze cathode reactions (Rozendal et al., 2008; Saheb-Alam et al., 2019b). No effect of biofilm formation could be seen on the CNP cathode, probably because they already had very high surface area and high catalytic activity.

### 4.2. Microbial community composition and function in the MECs

The anode, cathode, and suspension formed three distinct habitats with different microbial community composition in the MECs. In the suspension, microorganisms may have used carbon sources, hydrogen escaping the cathode biofilm, or cellular debris to sustain growth. The suspension had the highest similarity with the inoculum (Figure 4B), which could have been caused by sludge from the inoculum remaining in systems during the whole experiment. The suspension samples also had a high abundance of several methanogens including *Methanothrix, Ca Methanofastidiosum, Methanomassiliicoccus*, and *Methanolinea* (Supplementary Figure S11). *Methanothrix*, the most abundant methanogen in suspension samples, uses acetate as the sole source of energy (Patel and Sprott, 1990). This methanogen had very low abundance on the anode, where it was likely outcompeted by electrogens, and on the cathode, where it was outcompeted by hydrogenotrophic methanogens. In the suspension, there was also a large abundance of syntrophic bacteria such as *Syntrophomonas* and *Syntrophorhabdus*, as well as some species that seem to be hydrogen consuming such as *Hydrogenophaga*. Direct methane production from acetate by *Methanothrix* and fermentation of butyrate and propionate by syntrophs in association with hydrogenotrophic methanogens were likely the major functions in the suspension.

On the cathode, electrochemical generation of hydrogen was likely a major factor driving community assembly. Consequently, methanogens and *Acetobacterium* spp. were abundant. Methanogens in the *Methanobacteriaceae* family, which dominated the cathodes, are known for their use of hydrogen in the reduction of carbon dioxide (Enzmann et al., 2018). Similarly, *Acetobacterium* spp. use hydrogen and carbon dioxide in the production of acetic acid (Balch et al., 1977; Schuchmann and Müller, 2016). Other than these, an uncharacterized species from the *Peptostreptococcaceae* family (ASV14) was highly abundant on the carbon nanoparticle cathodes. Most of the species within this family are anerobic fermentative acetogens; therefore, this species could potentially be an acetogen as well (Pikuta et al., 2009; Gerritsen et al., 2014, 2018). The high abundance of hydrogen utilizing microorganisms on the cathode surface can explain why improved catalysis of the hydrogen evolution reaction was observed on the titanium and steel cathodes (Figure 3). The consumption of hydrogen lowers its partial pressure near the cathode surface, which makes hydrogen evolution more thermodynamically favorable (Philips, 2020). Extracellular hydrogenases adsorbed on the cathode surface can also improve catalysis (Deutzmann et al., 2015), and several hydrogenotrophic enrichment cultures have been shown to catalyze electrochemical hydrogen evolution (Saheb-Alam et al., 2019b). Due to the already large surface area of the carbon nanoparticles, the addition of a hydrogenotrophic culture did not result in an improvement of the hydrogen evolution.

On the anode, electrogenesis was the major metabolic function. As expected, species within *Deltaproteobacteria* were highly abundant. These are known electrogens associated with the microbial electrochemical systems (Bond and Lovley, 2003; Summers et al., 2010; Cao et al., 2019). Most of the other species were unknown, but it is highly probable that many of them have a fermentative metabolism based on the properties of the other genera found within the same family. For instance, bacteria from the *Macellibacteroides* genus were present on the anode surfaces. *Macellibacteroides* spp. are obligatory anerobic gram-positive bacteria that have a fermentative metabolism (Jabari et al., 2012). It has been seen that, in the presence of glucose, their fermentation process results in the production of acetate, butyrate, and isobutyrate (Jabari et al., 2012). Other species present, such as those from the *Anaerolineaceae* family, *Clostridiales* order, *Spirochaetaceae* family, and *Sedimentibacter* genus, have also been shown to produce compounds such as acetate or butyrate by fermentation (Menes and Muxí, 2002; Maune and Tanner, 2012; McIlroy et al., 2017). Most of these species need the presence of sugars for their fermentation process, resulting in the production of acetate. Even though sugars such as glucose were not present in the NM fed to the system, there is the possibility that polysaccharides found in the biofilm extracellular polymeric substances (EPS) could be a source for this process. A likely explanation for the slight increase in butyrate concentrations (Supplementary Figure S2) could be hydrogen generation occurring at the cathode, driving the butyrate production. It has been shown that biocathodes can drive the production of butyrate through the reduction in carbon dioxide (Ganigué et al., 2015; Tahir et al., 2021).

Uncharacterized species from the *Synergistaceae* and *Cloacimonadaceae* families were also observed on the anode (Supplementary Table S2). Both are known to be fermenters of organic acids and sugars. Research showed that species within the *Cloacimonadaceae* family are potentially syntrophic and could have the ability to produce compounds such as acetate and carbon dioxide from the fermentation of propionate under low hydrogen pressure (Schink, 1997; Ariesyady et al., 2007; Pelletier et al., 2008). Similar to *Cloacimonadaceae* spp., some of the species within *the Synergistaceae* family are syntrophic bacteria that produce hydrogen, carbon dioxide, and acetate as products of their fermentative metabolism in the presence of hydrogen-consuming microorganisms (Qiu et al., 2014). This potentially indicates a syntrophic interaction between these species and hydrogen- and acetate-consuming species, e.g., electrogens within *Deltaproteobacteria*. A positive interaction in the network analysis of the anodes was observed between the electrogen ASV1 and ASV9, which was classified as a putatively butyrate-oxidizing *Syntrophomonas* sp. This suggests that ASV9 oxidized butyrate to hydrogen and acetate, which were used by ASV1 to generate electrical currents.

### 4.3. Competition between electrogens on the anode

From the microbial correlation network analysis, it could be noted that most of the interactions seen on the cathode surfaces were positive, with only 7.6% of all interactions being negative. Since electrochemically generated hydrogen likely diffused throughout the environment surrounding the cathode, there is less competition to obtain hydrogen between the microorganisms in the cathode biofilm. In the anode biofilms, the negative interactions were 30.0% of all identified interactions. This indicates a higher degree of competition between the microorganisms present. Since the transfer of electrons require the microorganisms to have contact with the anode surface, this creates a situation where organisms with a similar function must compete for the limited space, resulting in one of the species winning and taking over. This can be illustrated by the negative correlation between ASV1 and a combination of ASV7 and ASV8. All three of these ASVs are *Deltaproteobacteria* spp. that most likely are electrogenic and are competing for the limited space available on the anode surface. ASV7 and ASV8 only differed by one base pair and were both classified as *Geobacter* sp. with the same species placeholder name in the Midas database (midas_s_9397). ASV1 differed from the other two by 14 or 15 bp and was an unclassified *Deltaproteobacteria* sp.

Malfunctioning CV tests led to gas formation at the electrode surface in S3, which caused foaming and inactivation of the electrogenic biofilm due to an increased over potential leading to the release of O_2_ and H_2_, thus disrupting the biofilm. O_2_ production on the anode may be caused by physically removing cells, poisoning strict anaerobes, and enabling a temporary bloom of O_2_-respiring microorganisms. H_2_ production on the cathode may both be due to feeding hydrogenotrophs and physically remove cells if bubbles are formed. The S3 foam sample had an almost identical microbial community composition to that of the other anode samples. ASV1 dominated in the foam sample but ASV8 dominated on the S3 anode at the end of the experiment. This indicates that the death of the microbial community on the anode surface during the foaming incident allowed for other bacteria present in the system to attach and increase in abundance. This could have been caused by the foaming incident creating a new environment on the anode selected for ASV8 rather than ASV1, or it could have been caused by stochastic factors.

The reason why different electrogenic ASVs dominated in different MECs is unclear. One possibility is random chance, which could be affected by the abundance in the initial inoculum (Supplementary Table S1). Based on the null model analysis with a diversity order of 0, many significant similarities could be established between samples for both the anode and the cathode. This means that the same bacteria are present in the systems. However, disruptive events such as the foaming incidents, differences in environmental conditions caused by minor uncontrollable differences in operation, and stochastic factors could have led to differences in their relative abundances. In addition to S3, which was exposed to a foaming incident, only the three MECs with carbon nanoparticle cathodes had high abundances of ASV7 or ASV8. This suggests that the cathode material could have had some influence on this subset of the anodic community. However, the effect of the cathode material is speculative, and no clear effect on the community as a whole could be observed.

Since the microbial community develops over time, the microbial analysis performed on the community present at the end of the experimental run might not reflect those present at different times during the experimental run. It is also important to take into consideration that the presence of a set of microbial taxa does not necessarily mean that the taxa are viable, and a way to address this would be to do stable isotope probing or RNA sequencing, which would allow for the determination of active and viable taxa.

In conclusion, the choice of cathode material did not cause significant differences in current generation or microbial community composition in the experiment. All MECs had a similar development with an initial lag phase without current generation lasting for 5–17 days followed by an increase in current density and coulombic efficiency that peaked around day 30 and then declined and reached steady levels. Stochastic initial colonization of the anodes led to differences in the lag time of the MEC. The microbial community of the suspension consisted mainly of fermenters. The cathode community was dominated by methanogens, while electrogens were found in high abundance in the anode microbial community. Competition between electrogenic taxa within *Deltaproteobacteria* on the anode, possibly influenced by stochastic initial colonization and disruptive events such as the foaming incidents, led to different taxa dominating on different anodes. Competitive interactions were much more prominent in the anode communities in comparison to the cathode communities.

## Data availability statement

The datasets presented in this study can be found in online repositories. The names of the repository/repositories and accession number(s) can be found in the article/Supplementary material.

## Author contributions

MA: design of experiment, performance of laboratory work, data analysis, and writing. MR: data analysis and writing. OM: design of experiment and writing. FP, B-MW, and AF: contributed to the writing and revision. All authors contributed to the article and approved the submitted version.
