# Supplementary material for: Evidence of competition between electrogens shaping electroactive microbial communities in microbial electrolysis cells
Source: Front Microbiol. 2022 Dec 16;13:959211. doi: 10.3389/fmicb.2022.959211 (PMC9800620; doi:10.3389/fmicb.2022.959211)
Supplement: Supplementary file 2 [file Data_Sheet_2.docx]

**Supplementary material**


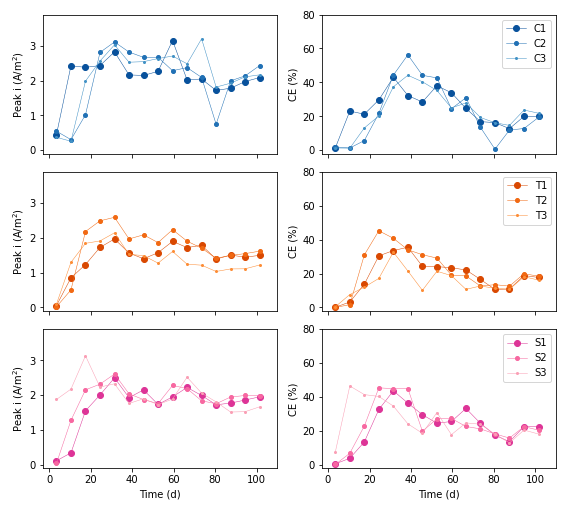


**Figure S1.** Peak current density (i) and coulombic efficiency (CE) during the experiment. Weekly values are shown.


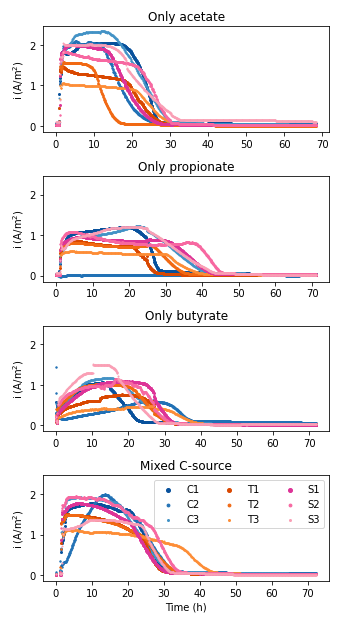


**Figure S2.** Current generation profiles during the special tests with only one carbon source. The lowest panel shows the current profiles with all three carbon sources present in the nutrient broth.


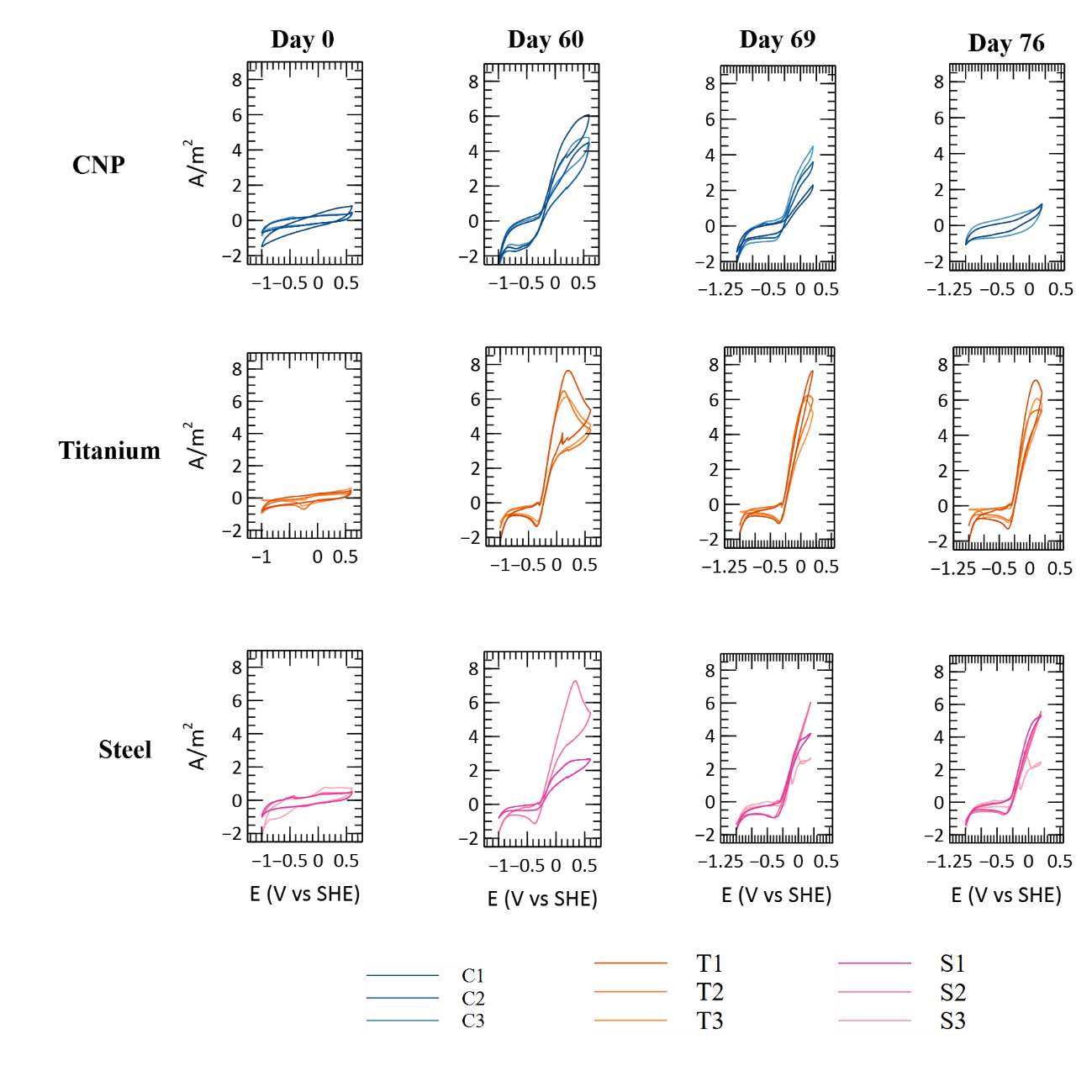


**Figure S3.** Cyclic voltammetry measurements for all anodes. C2 is missing on day 76 and S3 is missing on day 60 because of malfunctioning reference electrodes.


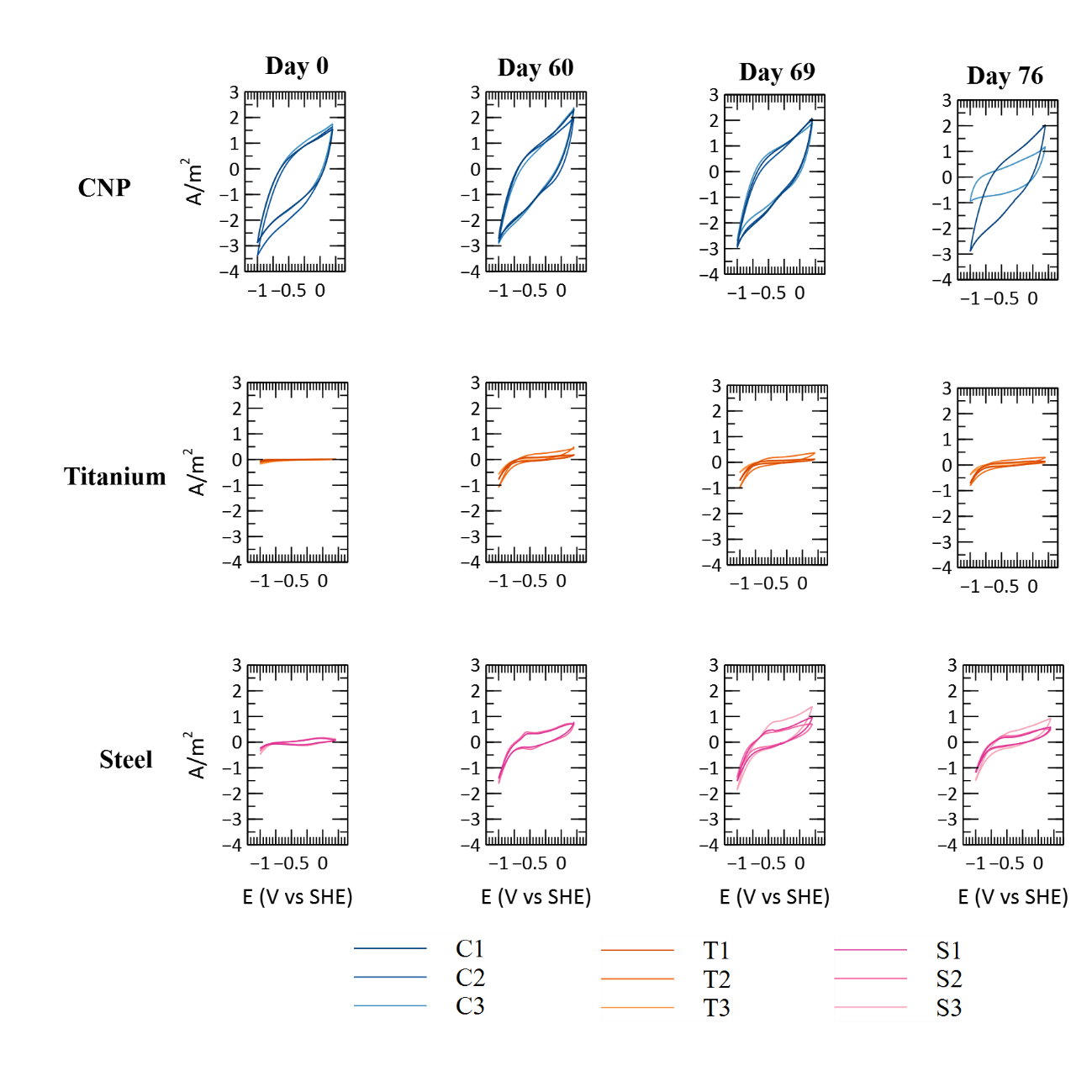


**Figure S4.** Cyclic voltammetry measurements for all cathodes. C2 is missing on day 76 and S3 is missing on day 60 because of malfunctioning reference electrodes.


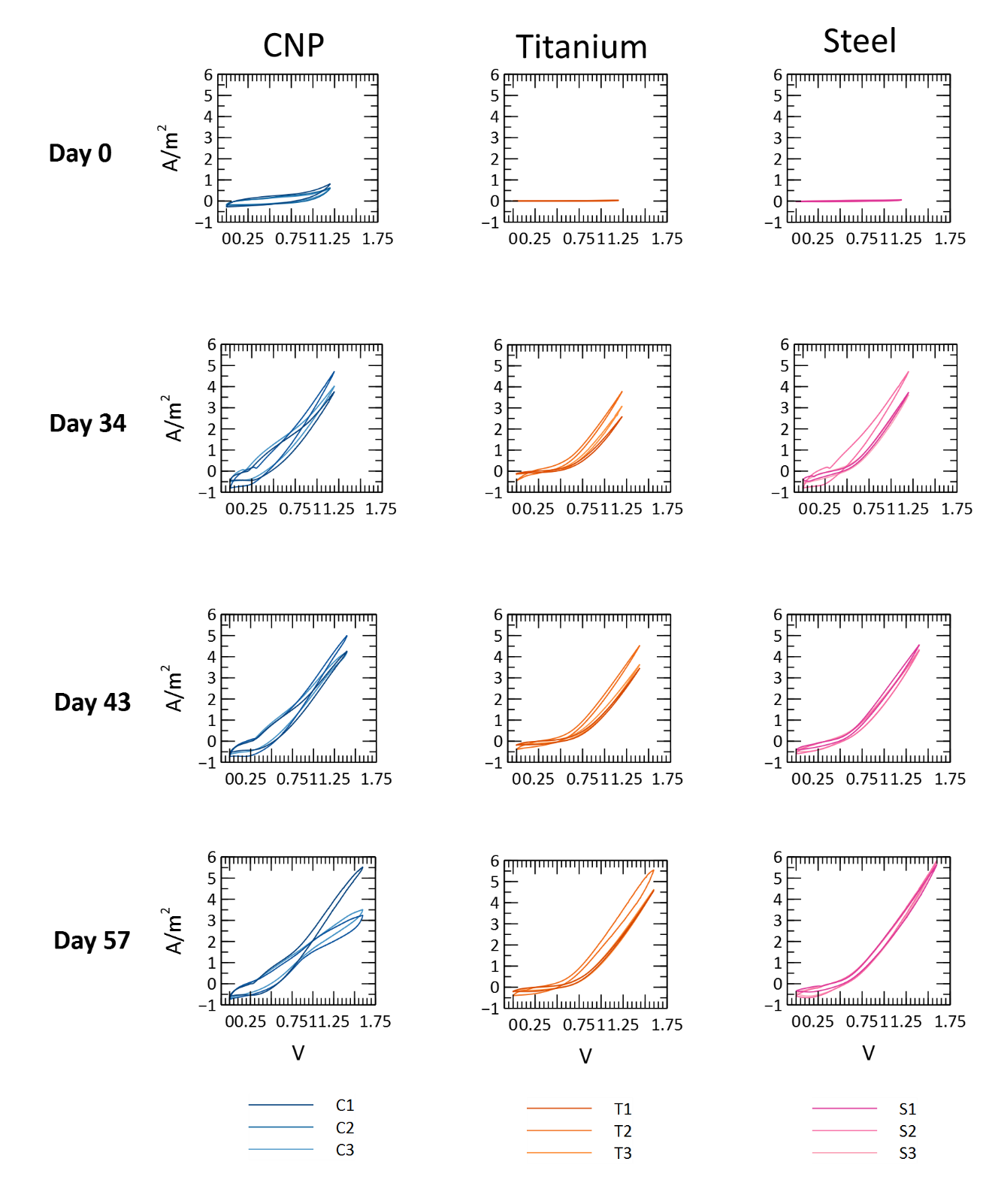


**Figure S5.** Polarization curves from different time point during the experimental run.


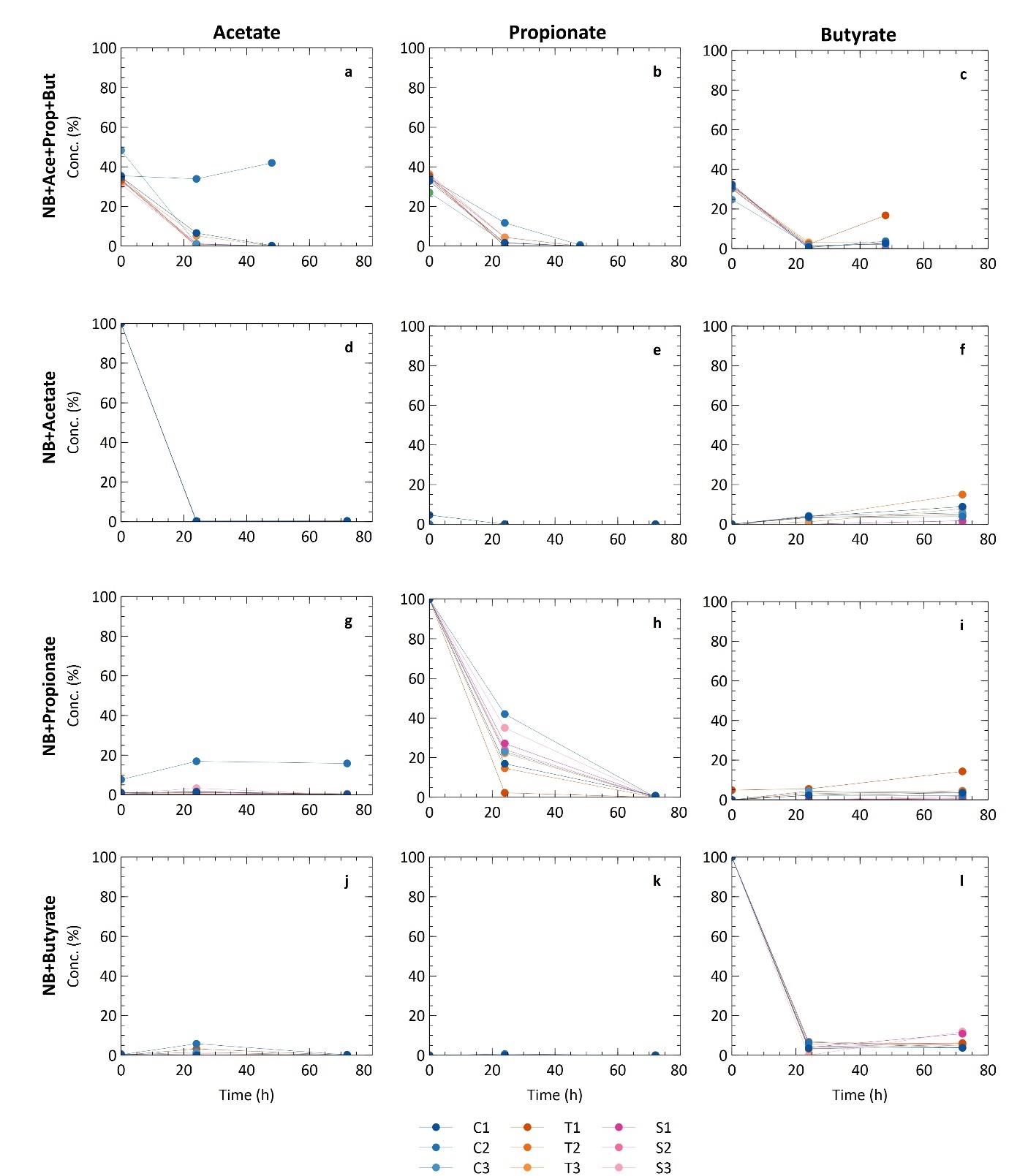


**Figure S6.** Changes in the acetate, propionate, and butyrate concentrations during the special tests with different carbon source compositions. **(a-c)** Acetate, propionate, and butyrate concentrations when all three carbon sources were added simultaneously. **(d-f)** Concentrations when only acetate was added. **(g-i)** Concentrations when only propionate was added. **(j-l)** Concentrations when only butyrate was added. C1-C3 are MECs with carbon nanoparticle cathodes, T1-T3 have titanium cathodes, and S1-S3 have steel cathodes. The y-axis shows the concentration as a percentage of the total reducing equivalent concentration in the NB (175 mmol e^-^/L). Acetate, propionate and butyrate have been abbreviated to Ace, Prop, and But, respectively.


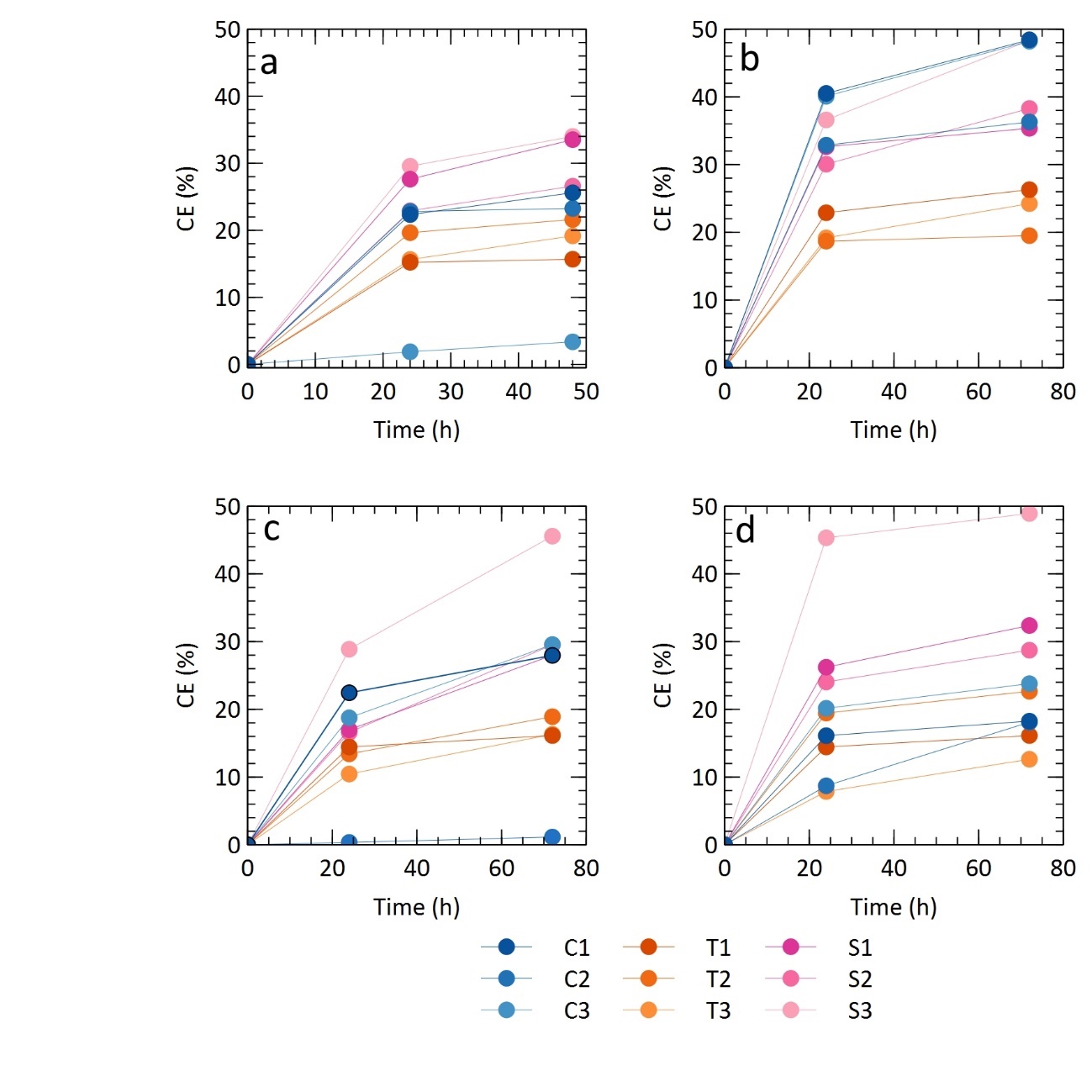


**Figure S7.** Coulombic efficiencies in the tests with single carbon sources. **(a)** Test with all three carbon sources (acetate, propionate, and butyrate). **(b)** Only acetate added. **(c)** Only propionate added. **(d)**  Only butyrate added. C1-C3 are MECs with carbon nanoparticle cathodes, T1-T3 have titanium cathodes, and S1-S3 have steel cathodes.


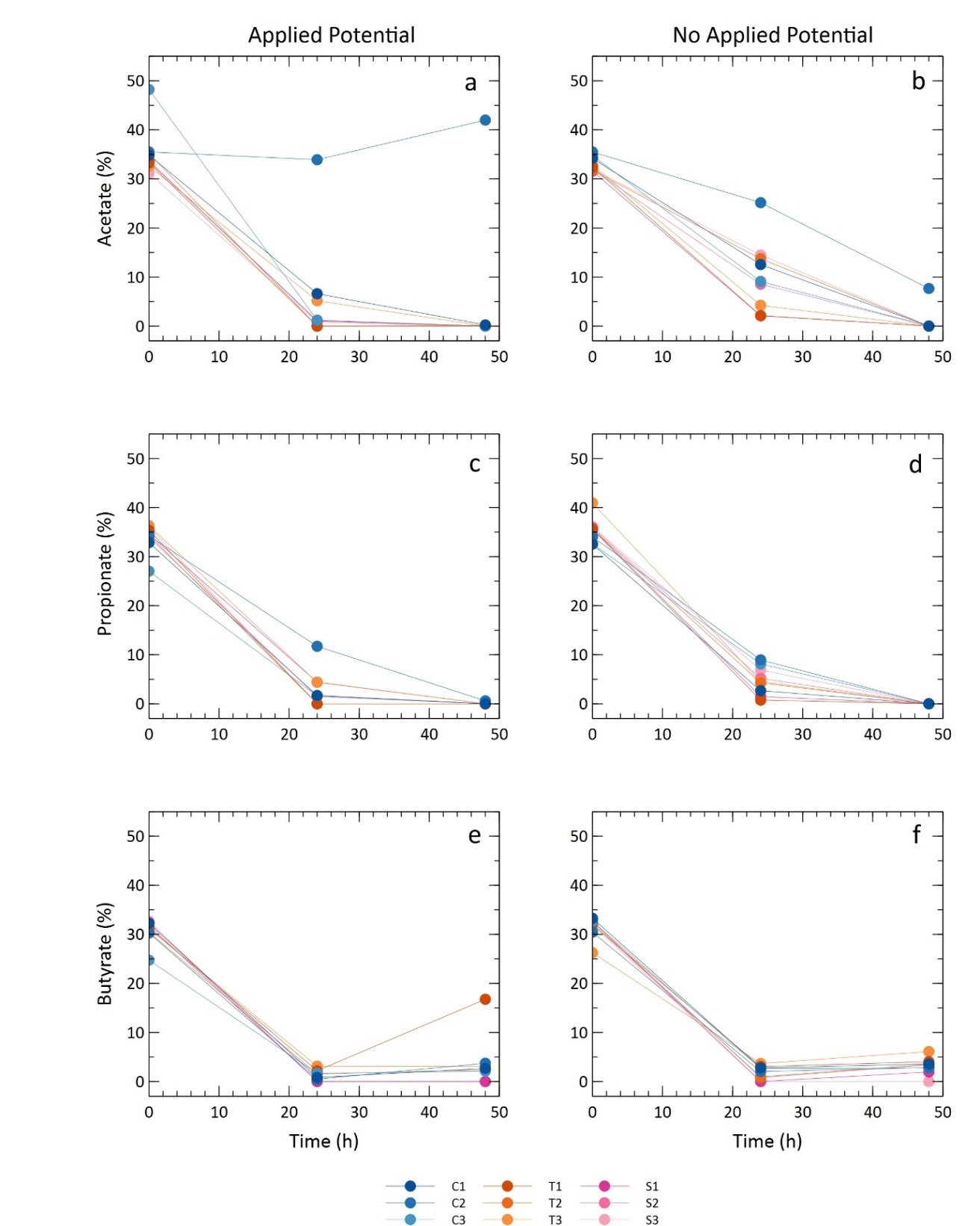


**Figure S8.** Acetate, propionate, and butyrate concentration over time in the presence and absence of an applied potential on the system. NB with all three carbon sources (acetate, propionate, and butyrate) was added to the MECs and the consumption pattern of these substrates was measured over time. The y-axis shows the reducing equivalent associated with the carbon source as a percentage of the total reducing equivalent of all carbon sources. The columns show presence **(a, c, e)** and absence **(b, d, f)** of an applied potential. The rows show the concentrations of the carbon sources acetate **(a, b)**, propionate **(c, d)**, and butyrate **(e, f)**. C1-C3 are MECs with carbon nanoparticle cathodes, T1-T3 have titanium cathodes, and S1-S3 have steel cathodes.


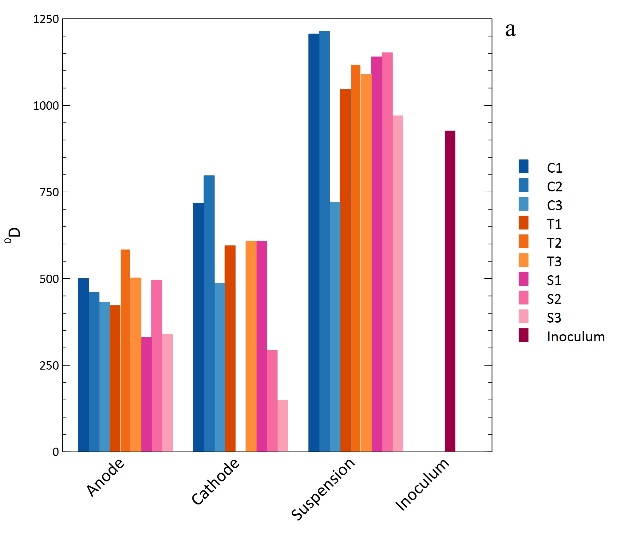

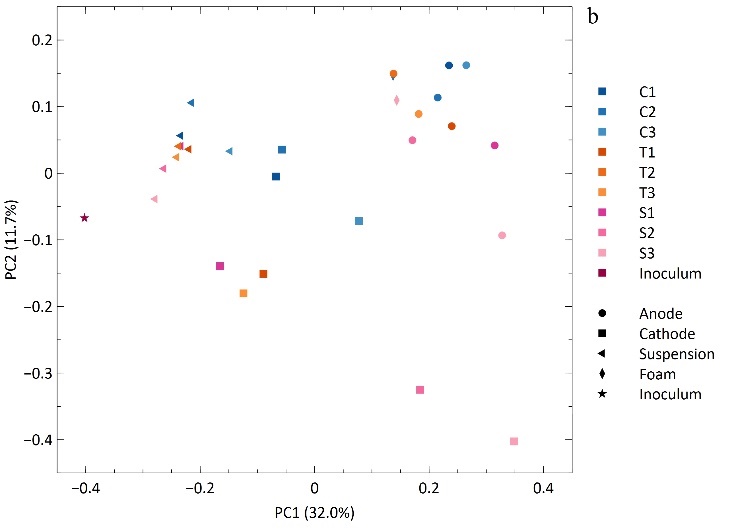


**Figure S9. (a)** Bar graph with the alpha diversity values for all samples with a diversity order of 0. **(b)** Principal coordinate analysis made from the dissimilarity matrix calculated with a diversity order of 0.


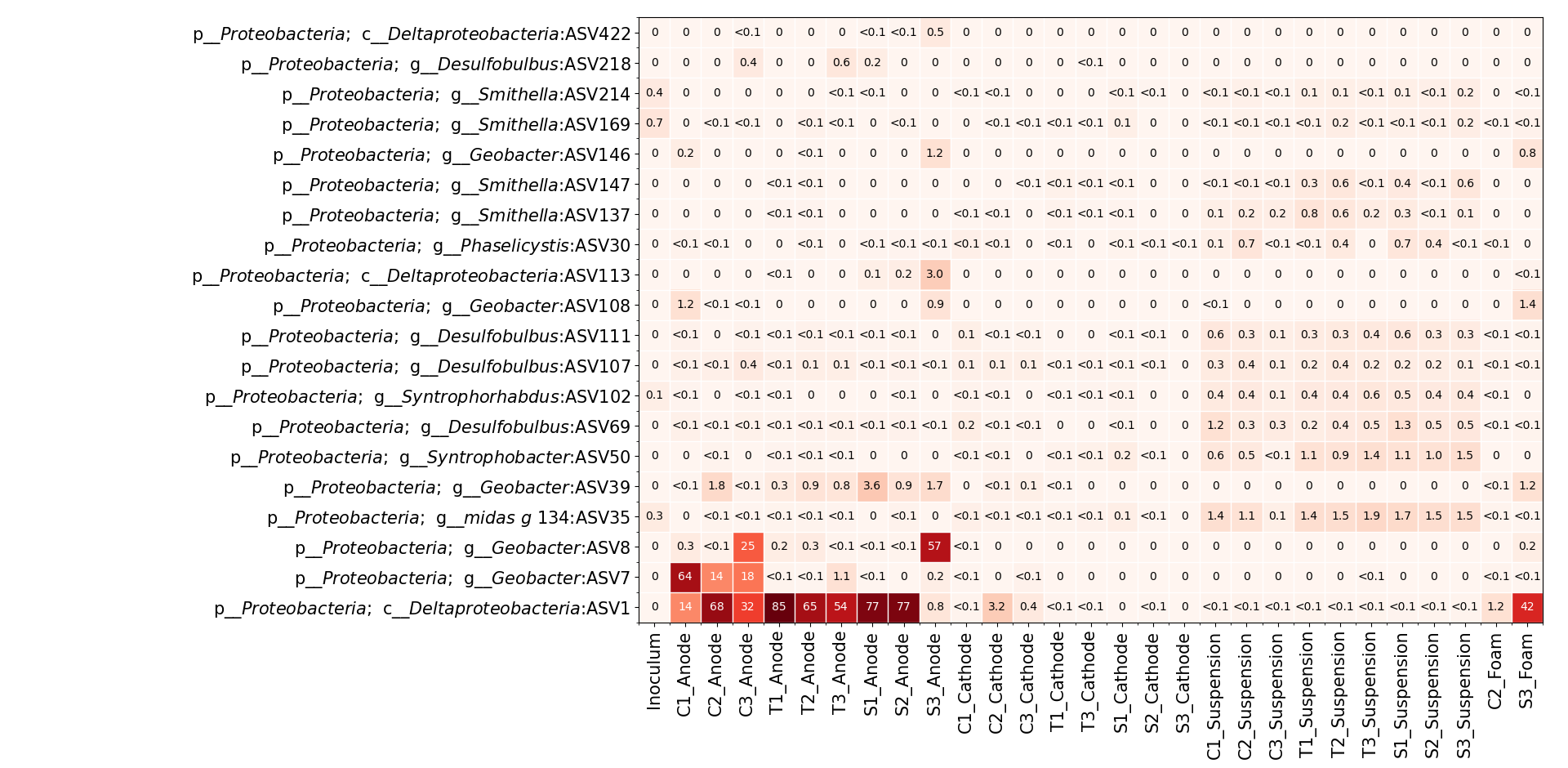


**Figure S10.** Heatmap depicting the relative abundance of the top 20 most abundant taxa within the *Deltaproteobacteriae* sp. present in the 9 reactors, the inoculum as well as the foam samples from C2 and S3. The T2 cathode was excluded due to low number of reads.


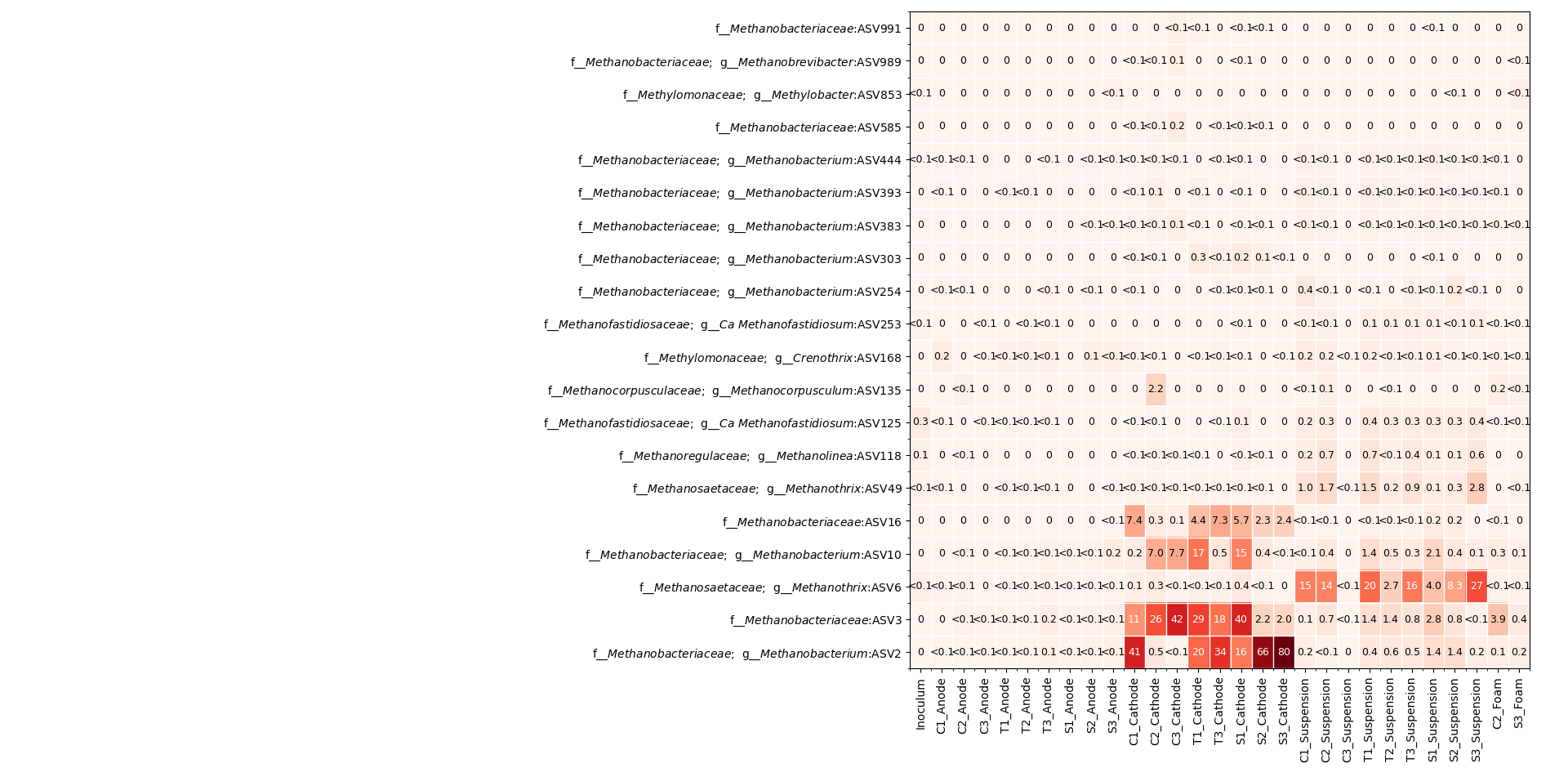


**Figure S11.** Heatmap depicting the relative abundance of the top 20 most abundant taxa involved in methanogenesis or methanotrophy (only ASV168) in the 9 reactors, the inoculum as well as the foam samples from C2 and S3. The T2 cathode was excluded due to low number of reads.


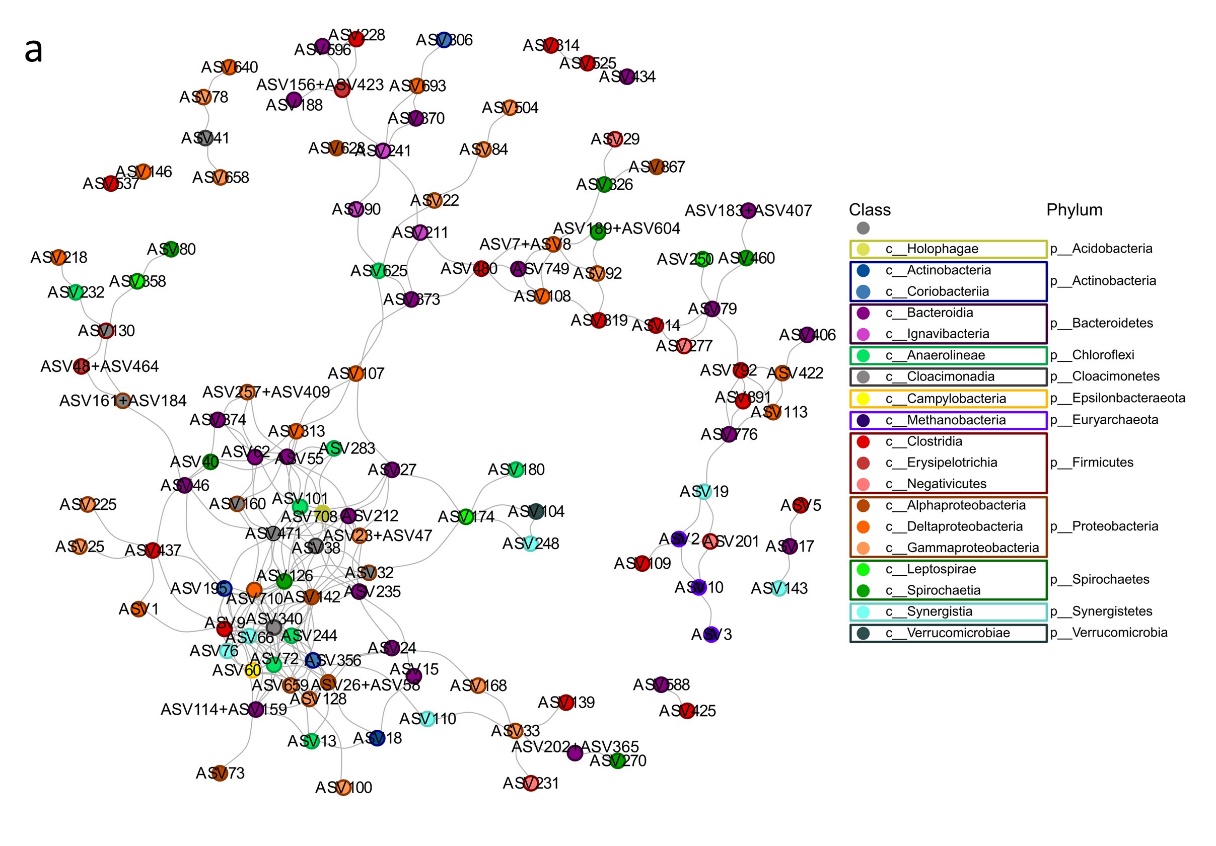


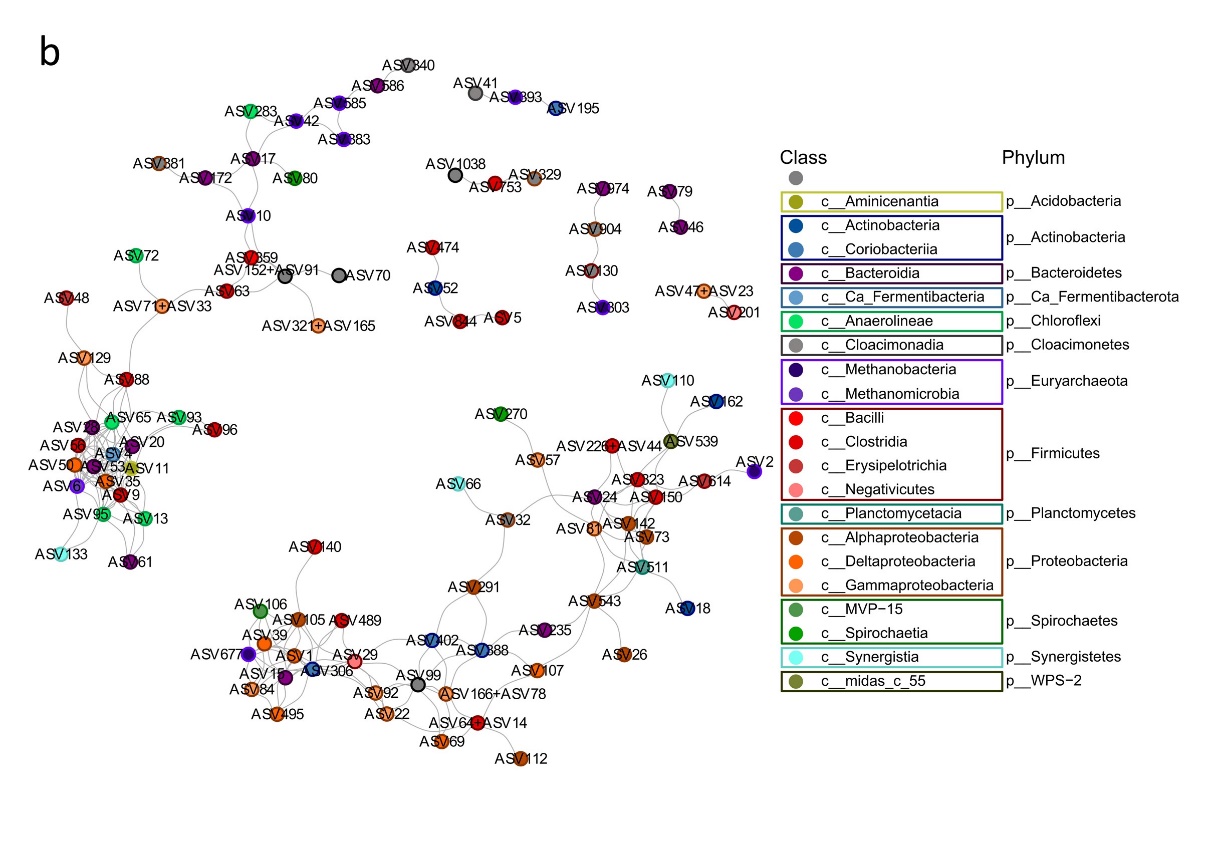


**Figure S12.** Microbial correlation network depicting the positive interactions. **a)** The anode microbial community. **b)** The cathode microbial community. Colours depict taxonomy of each ASV at the class level.

**Table S1.** Tables depicting the Raup-Crick null model results for the anode and cathode for a diversity order (q) 0 and 1. Significant dissimilarities (>0.95) and significant similarities (<0.05) are marked with red and blue respectively.

Anode, q=0

|  | C2 | C3 | T1 | T2 | T3 | S1 | S2 | S3 |
| --- | --- | --- | --- | --- | --- | --- | --- | --- |
| C1 | 0.16 | 0.00 | 0.00 | 0.82 | 0.03 | 0.00 | 0.73 | 0.04 |
| C3 |  | 0.00 | 0.00 | 0.96 | 0.46 | 0.00 | 0.82 | 0.05 |
| C3 |  |  | 0.00 | 0.03 | 0.00 | 0.00 | 0.20 | 0.39 |
| T1 |  |  |  | 0.00 | 0.00 | 0.00 | 0.00 | 0.00 |
| T2 |  |  |  |  | 0.13 | 0.00 | 0.05 | 0.98 |
| T3 |  |  |  |  |  | 0.00 | 0.00 | 0.63 |
| S1 |  |  |  |  |  |  | 0.00 | 0.00 |
| S2 |  |  |  |  |  |  |  | 0.37 |

Anode, q=1

|  | C2 | C3 | T1 | T2 | T3 | S1 | S2 | S3 |
| --- | --- | --- | --- | --- | --- | --- | --- | --- |
| C1 | 0.65 | 0.62 | 0.89 | 0.94 | 0.83 | 0.80 | 0.93 | 1.00 |
| C3 |  | 0.58 | 0.38 | 0.67 | 0.64 | 0.30 | 0.54 | 0.99 |
| C3 |  |  | 0.57 | 0.68 | 0.63 | 0.49 | 0.63 | 0.55 |
| T1 |  |  |  | 0.28 | 0.49 | 0.04 | 0.08 | 1.00 |
| T2 |  |  |  |  | 0.58 | 0.19 | 0.34 | 0.99 |
| T3 |  |  |  |  |  | 0.26 | 0.53 | 0.96 |
| S1 |  |  |  |  |  |  | 0.08 | 0.87 |
| S2 |  |  |  |  |  |  |  | 1.00 |

Cathode, q=0

|  | C2 | C3 | T1 | T3 | S1 | S2 | S3 |
| --- | --- | --- | --- | --- | --- | --- | --- |
| C1 | 0.94 | 0.00 | 0.60 | 0.12 | 0.38 | 0.00 | 0.00 |
| C2 |  | 0.18 | 0.28 | 1.00 | 1.00 | 0.06 | 0.04 |
| C3 |  |  | 0.10 | 0.05 | 0.48 | 0.00 | 0.00 |
| T1 |  |  |  | 0.05 | 0.06 | 0.00 | 0.00 |
| T3 |  |  |  |  | 0.00 | 0.00 | 0.00 |
| S1 |  |  |  |  |  | 0.00 | 0.38 |
| S2 |  |  |  |  |  |  | 0.00 |

Cathode, q=1

|  | C2 | C3 | T1 | T3 | S1 | S2 | S3 |
| --- | --- | --- | --- | --- | --- | --- | --- |
| C1 | 0.99 | 0.93 | 0.81 | 0.63 | 0.81 | 0.26 | 0.08 |
| C2 |  | 0.42 | 0.86 | 0.97 | 0.89 | 0.81 | 0.60 |
| C3 |  |  | 0.60 | 0.83 | 0.55 | 0.76 | 0.58 |
| T1 |  |  |  | 0.56 | 0.20 | 0.43 | 0.25 |
| T3 |  |  |  |  | 0.57 | 0.13 | 0.06 |
| S1 |  |  |  |  |  | 0.53 | 0.33 |
| S2 |  |  |  |  |  |  | 0.00 |
